# Sustained COVID-19 community transmission and potential super spreading events at neglected afro-ecuadorian communities assessed by massive RT-qPCR and serological testing of community dwelling population

**DOI:** 10.3389/fmed.2022.933260

**Published:** 2022-08-18

**Authors:** Alexander Paolo Vallejo-Janeta, Diana Morales-Jadan, Maria Belen Paredes-Espinosa, Barbara Coronel, Heberson Galvis, Hugo Renato Bone-Guano, Belen Amador Rodriguez, Guadalupe Gomez Abeledo, Byron Freire-Paspuel, Esteban Ortiz-Prado, Ismar Rivera-Olivero, Aquiles Rodrigo Henriquez-Trujillo, Tannya Lozada, Miguel Angel Garcia Bereguiain, the UDLA COVID-19 Team, Tatiana Jaramillo

**Affiliations:** ^1^One Health Research Group, Universidad de Las Américas, Quito, Ecuador; ^2^“UDLA-COVID-19 Team”, Universidad de Las Américas, Quito, Ecuador; ^3^Universidad Técnica Luis Vargas Torres, Esmeraldas, Ecuador; ^4^Decanato de Investigación y Vinculación, Universidad de Las Américas, Quito, Ecuador

**Keywords:** SARS-CoV-2, COVID-19, Ecuador, seroprevalence, RT-qPCR, Afro-Ecuadorian population, Esmeraldas, rural communities

## Abstract

**Background:**

Neglected ethnic minorities from underserved rural populations in Latin America are highly vulnerable to coronavirus disease 2019 (COVID-19) due to poor health infrastructure and limited access to severe acute respiratory syndrome coronavirus 2 (SARS-CoV-2) diagnosis. Esmeraldas is a mainly rural province of the Coastal Region of Ecuador characterized by a high presence of Afro-Ecuadorian population living under poverty conditions.

**Objective:**

We herein present a retrospective analysis of the surveillance SARS-CoV-2 testing in community-dwelling population from Esmeraldas carried out by our university laboratory in collaboration with regional health authorities during the first week of October 2020, in a region where no public SARS-CoV-2 detection laboratory was available at that time.

**Results:**

A total number of 1,259 people were tested for SARS-CoV-2 by Reverse Transcription quantitative Polimerasa Chain Reaction (RT-qPCR), resulting in an overall infection rate of 7.7% (97/1259, 95% *CI*: [6.32–9.35%]) for SARS-CoV-2, up to 12.1% in some communities. Interestingly, community-dwelling super spreaders with viral loads over 10^8^ copies/ml represented 6.2% of the SARS-CoV-2-infected population. Furthermore, anti-SARS-CoV-2 IgG serological tests were applied to the same study group, yielding an overall seroprevalence of 11.68% (95% *CI*: [9.98–13.62%]) but as high as 24.47% at some communities.

**Conclusion:**

These results support active COVID-19 community transmission in Esmeraldas province during the first semester of the COVID-19 pandemic as it has been shown for other rural communities in the Ecuadorian Coastal Region.

## Introduction

The outbreak of coronavirus disease 2019 (COVID-19) raised concerns in the global scientific and health communities since the first 27 cases were reported in December 2019 from Wuhan, China (1). The severe acute respiratory syndrome coronavirus 2 (SARS-CoV-2) spread readily and quickly around the world, WHO declared a global pandemic, and the first cases in Latin America were reported just 2 months after the original report (1, 2). Until March 2022, more than 480 million COVID-19 cases and more than 6 million deaths were reported worldwide (2). In Ecuador, more than 830,000 cases and 35,000 deaths associated to COVID-19 were reported since the arrival of the first case in February 2020 till August 9 2021 (2)^1^ From the early stages of the COVID-19 pandemic, a wide variety of recommendations were handed by the World Health Organization (WHO) like using mask, social distancing, and isolation of confirmed cases to slow down the spread of the disease. Moreover, there was a permanent call on the public media from WHO authorities asking to governments worldwide to carry as many tests as possible as the best strategy to control the virus spread.

The COVID-19 pandemic hits all health systems worldwide. However, its impact was particularly greater in low- and middle-income settings, such as those found in developing countries from Latin America (3, 4). Despite the latter arrival of the pandemic to these countries, and the subsequent time they had to prepare and prevent an eventual health system collapse, the saturation of hospitals and shortage of supplies were inevitable (5). These lead to a public health crisis as seen during March–April 2020 in Guayaquil, the most populated city of Ecuador (4–8). At the beginning of this emergency, only the National Institute of Research in Public Health (INSPI) laboratories, located in the 3 main cities of Ecuador (Guayaquil, Quito, and Cuenca) performed SARS-CoV-2 detection using RT-qPCR within the public health system, which was translated in a poor testing ratio of 7.46 PCR tests per 10,000 people, and one of the highest COVID-19 mortality rates (10.93 deaths per million people) in Latin America (7). During the first 18 months of the COVID-19 pandemic, less than 2 million SARS-CoV-2 RT-qPCR tests have been done for a 17 million Ecuadorian population, with a positivity rate over 20%, according to the Ecuadorian Ministry of Health (MoH) (9). This is clearly higher that the 5% positivity rate recommended for the WHO and it means that SARS-CoV-2 testing in Ecuador was not enough for an effective control and prevention strategy (10).

Esmeraldas is located in the Ecuadorian Northern Coastal Region; it is the seventh largest province on the surface and the eighth most populated one with 491,168 inhabitants, mostly distributed across rural communities in seven cantons; and it is one of the poorest regions in the country (11–13). Additionally, 43% of its population identifies themselves as Afro-Ecuadorian, making Esmeraldas the main Afro-Ecuadorian region of Ecuador (13). According to MoH, during the first semester of the COVID-19 pandemic (up to September 12, 2020), with a total of 9,129 SARS-CoV-2 RT-qPCR tests done, less than 2% of Esmeralda's population (185.9 test per 10,000 inhabitants) was tested despite the dramatic 39.8% positivity rate reported (14).

This study aims to insight into the epidemiological situation of Esmeraldas province in Ecuador during the first wave of the COVID-19 pandemic, where community transmission was suspected to happen for months according to the media reports of public health authorities. We present the retrospective analysis of a surveillance SARS-CoV-2 testing intervention during October 2020, carried out by Universidad de Las Américas (UDLA) in coordination with local community leaders, our colleagues from “Universidad Técnica Luis Vargas Torres” and the provincial government (“Prefectura de Esmeraldas”).

## Methods

### Study design and setting

**Figure 1:**
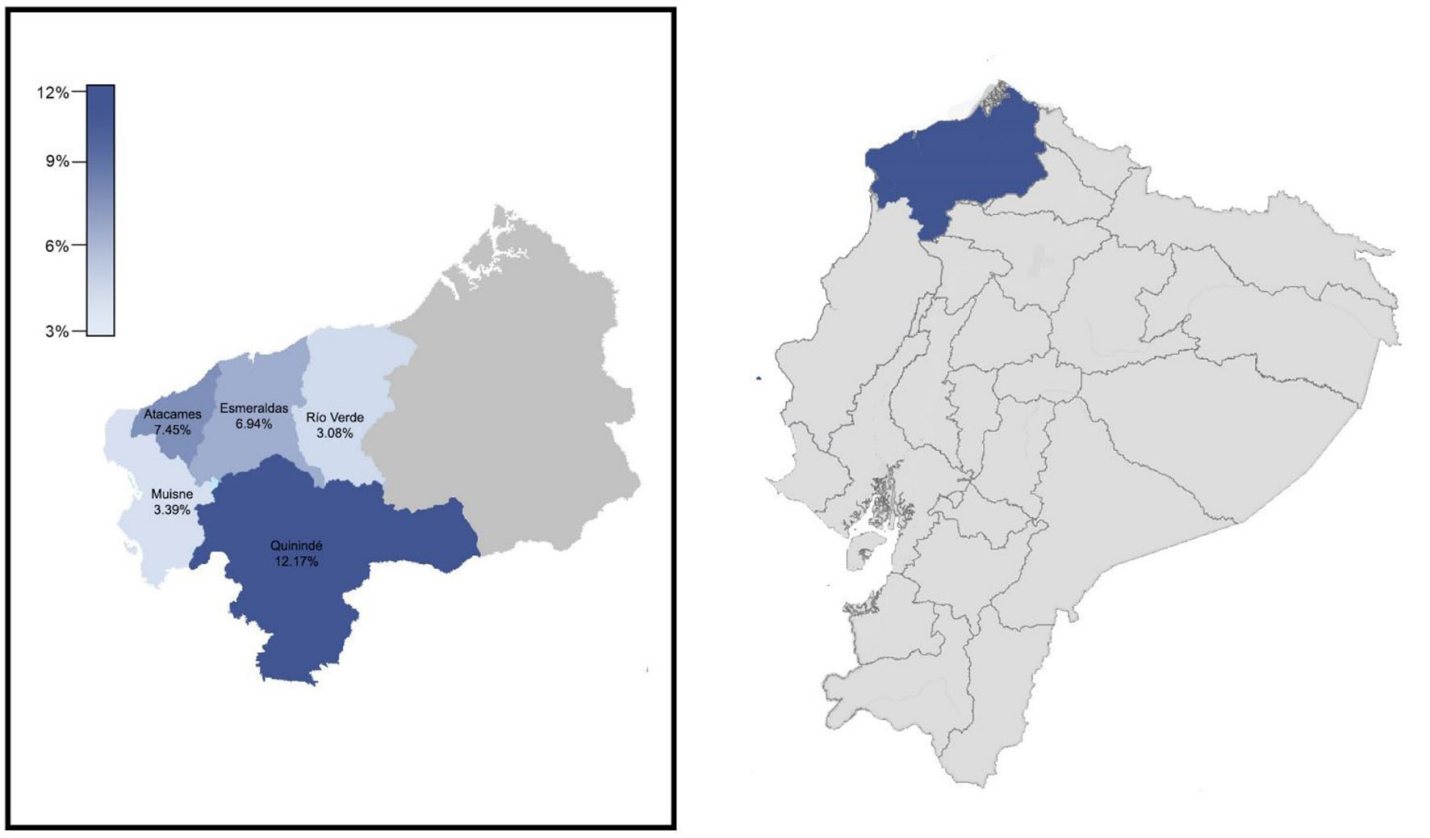
Location of esmeraldas province within ecuador. Cantons included in the surveillance carried out during October 2020 and their severe acute respiratory syndrome coronavirus 2 (SARS-CoV-2) infection rates by RT-qPCR.

A total number of 1,259 individuals enrolled in this surveillance. All samples were taken from community-dwelling asymptomatic or mild symptomatic individuals at the communities visited from 12th to 19th October 2020 in five out of seven cantons of Esmeraldas province: Esmeraldas, Atacames, Muisne, and Quinindé y Rio Verde (Figure 1). Two cantons were not included in the study (San Lorenzo and Eloy Alfaro) for logistic and security reasons, although those two cantons share the same demographic and socioeconomic features with the five cantons tested. Esmeraldas (capital city of the province with 150,000 inhabitants approximately) is the only canton, such as truly urban communities, where 5 different locations within the city were included for a total of 778 individuals tested. The other 4 cantons are rural ones, and 481 samples out of 7 different communities were included (3 communities for Quininde, 2 communities for Atacames, and 1 community for Muisne and Rio Verde). Although socioeconomic and ethnic information was not collected, the majority of the population enrolling the surveillance was low-income Afro-Ecuadorians.

As the study is a retrospective analysis of the data collected during a COVID-19 surveillance intervention as part of an aid program funded by “Fondo Salvar Vidas” from Banco de Guayaquil, to provide free access to SARS-CoV-2 testing, the communities were selected at convenience following the recommendations from the local organizations and the provincial government of Esmeraldas responsible for the logistics of this surveillance program.

### Sample collection, RNA extraction and RT-qPCR for SARS-CoV-2 diagnosis using the CDC protocol

Nasopharyngeal swabs were collected on 0.5 ml TE pH 8 buffer for SARS-CoV-2 diagnosis by RT-qPCR following an adapted version of the CDC protocol by using PureLink Viral RNA/DNA Mini Kit (Invitrogen, USA) as an alternate RNA extraction method and CFX96 BioRad instrument (15–22). Briefly, the CDC-designed RT-qPCR FDA EUA 2019-nCoV CDC kit (IDT, USA) is based on N1 and N2 probes to detect SARS-CoV-2 and RNase P as an RNA extraction quality control (21, 22). Also, negative controls (TE pH 8 buffer) were included as a control for carryover contamination, one for each set of RNA extractions, to guarantee that only true positives were reported. For viral loads calculation, the 2019-nCoV N positive control (IDT, USA) was used, provided at 200.000 genome equivalents/μl, and a factor of 200 was applied to convert the viral loads to genome equivalents/ml and then converted to a logarithmic scale. The individuals with viral loads bigger than 10^8^ copies/ml are considered SARS-CoV-2 super spreaders as it has been previously described (23).

### Serological test for anti SARS-CoV-2 IgG

A commercially available lateral-flow immunochromatographic anti-SARS-CoV-2 IgG from INNOVITA (TANGSHAN Biological Technology Co. Ltd, Hebei, China) was used. The appropriate sample volume (20 ul of venous whole blood) was transferred to the indicated sample port, followed immediately by provided diluent, following manufacturer instructions. The lateral flow cartridges were incubated for 15 min at room temperature before readings. Results negative or positive according to manufacturer instructions in each cartridge were read for test line intensity by two independent readers blinded to specimen status.

According to the manufacturer manual, the expected sensitivity and specificity for the test are 87.3 and 100%, respectively. We carried out an internal evaluation with 127 serum samples from SARS-CoV-2 positive individuals by RT-qPCR and 40 serum samples prior to the COVID-19 pandemic, and we got a sensitivity of 79.5% (95% CI: 71.5–86.2%) and specificity of 100% (95% *CI*: 91.2–100%) (24).

### Statistical analysis

For the statistical analysis of data, positivity rates and viral loads were calculated for each canton, as well as for different age groups, sex, and COVID-19 symptoms status. Seroprevalences were also calculated for all cantons. To assess differences in the positivity rates and viral loads, a non-parametric statistical test (Wilcoxon and Kruskal–Wallis) for comparison of proportions was applied. Confident intervals were calculated for a significance of 95%. All statistical analysis was carried out using R software.

*Ethical approval and consent to participate*. All participants signed informed consent to participate freely and voluntarily in this SARS-CoV-2 testing surveillance. This study is a secondary analysis of the anonymized laboratory results from a previous surveillance testing done in the context of the COVID-19 pandemic. Nevertheless, the study was approved by the Institutional Review Board from Hospital General San Francisco (Quito) with code CEISH-HGSF-2021-002.

## Results

### SARS-CoV-2 infection surveillance by RT-qPCR

A total of 1,259 samples for SARS-CoV-2 detection by RT-qPCR were taken in Esmeraldas province, distributed along five cantons: Esmeraldas, Rio Verde, Quininde, Muisne, and Atacames (Figure 1; Supplementary Table 1). The population was evenly distributed between men and women. Most of the individuals were adults (mean = 41.26 ± 0.48 years). The overall SARS-CoV-2 infection rate found in the province was 7.71% (97/1259, 95% *CI*: [6.32–9.35%]). See Supplementary Table 1 for details. The distribution of SARS-CoV-2 RT-qPCR individuals by sex and age is detailed in Supplementary Figure 2. The average age for SARS-CoV-2 positive individuals was 42.11 ± 1.81 years old. The SARS-CoV-2 infection rates for men and women were 6.35% (39/614, 95% *CI*: [4.61–8.66%]) and 8.99% (58/645, 95% *CI*: [6.95–11.54%]), respectively; although this difference was not significant (*p*-value > 0.05). SARS-CoV-2 prevalence varies among age groups, with values of 4.23% (3/71, 95% *CI*: [1.10–12.67%]) for children (0–14 years), 7.91% (44/556, 95% *CI*: [5.87–10.56%]) for young adults (14–39 years), 7.16% (31/433, 95% *CI*: [4.99–10.11%]) for adults (40–60 years), and 9.55% (19/199, 95% *CI*: [5.99–14.72%]) for elders (>60 years). Nevertheless, neither significant differences nor trends were found between each age group and prevalence of SARS-CoV-2 infection.

The five cantons included in the study were divided into two categories: rural and urban. All the communities from Atacames, Muisne, Rio Verde, and Quininde were rural and 43 SARS-CoV-2 positive individuals out of 481 ones tested were found, yielding an attack rate of 8.94% (95% *CI*: [6.61–11.94%]). All the communities for Esmeraldas canton were urban, and 54 SARS-CoV-2 positive individuals out of 778 ones tested were found, yielding an infection rate of 6.94% (95% *CI*: [5.30–8.66%]). Those values were not statistically different (*p*-value > 0.05). There are significant differences (*p*-value <0.01) in the SARS-CoV-2 infection rates among the cantons included in the study (Figure 1; Table 1): 7.45% (7/94, 95% *CI*: [3.30–15.24]) for Atacames, 6.94% (54/778, 95% *CI*: [5.30–9.02]) for Esmeraldas, 3.39% (2/59, 95% *CI*: [0.59–12.75]) for Muisne, 12.17% (32/263, 95% *CI*: [8.59–16.89]) for Quininde, and 3.08% (2/65, 95% *CI*: [0.54–11.64]) for Rio Verde.

**Table 1:** Severe acute respiratory syndrome coronavirus 2 (SARS-CoV-2) infection rate by RT-qPCR and anti-SARS-CoV-2 Ig G seroprevalence for the 5 cantons included on this surveillance study at Esmeraldas province in October 2020.

**CANTON**	**SARS-CoV-2 infection rate (%)**	**SARS-CoV-2 infection rate per 10 000 inhabitants**	anti-SARS-CoV-2 IgG seroprevalence (%)*	**anti-SARS-CoV-2 IgG seroprevalence per 10 000 inhabitants**	**Percentage of population sampled**
Esmeraldas	6.94 [5.30–9.02]	2.85	11.10 [9.02–13.57]	4.54	0.41
Quininde	12.17 [8.59–16.89]	2.61	12.21 [8.62–16.95]	2.61	0.22
Atacames	7.45 [3.30–15.24]	1.69	24.47 [16.44–34.61]	5.54	0.23
Muisne	3.39 [0.59–12.75]	0.70	6.78 [2.19–17.27]	1.41	0.21
Rio Verde	3.08 [0.54–11.64]	0.74	1.67 [0.09–10.14]	0.37	0.24
Province overall	7.71 [6.32–9.35]	1.82	11.68 [9.98–13.62]	2.73	0.24

### SARS-CoV-2 viral load distribution and super spreaders prevalence

**Figure 2:**
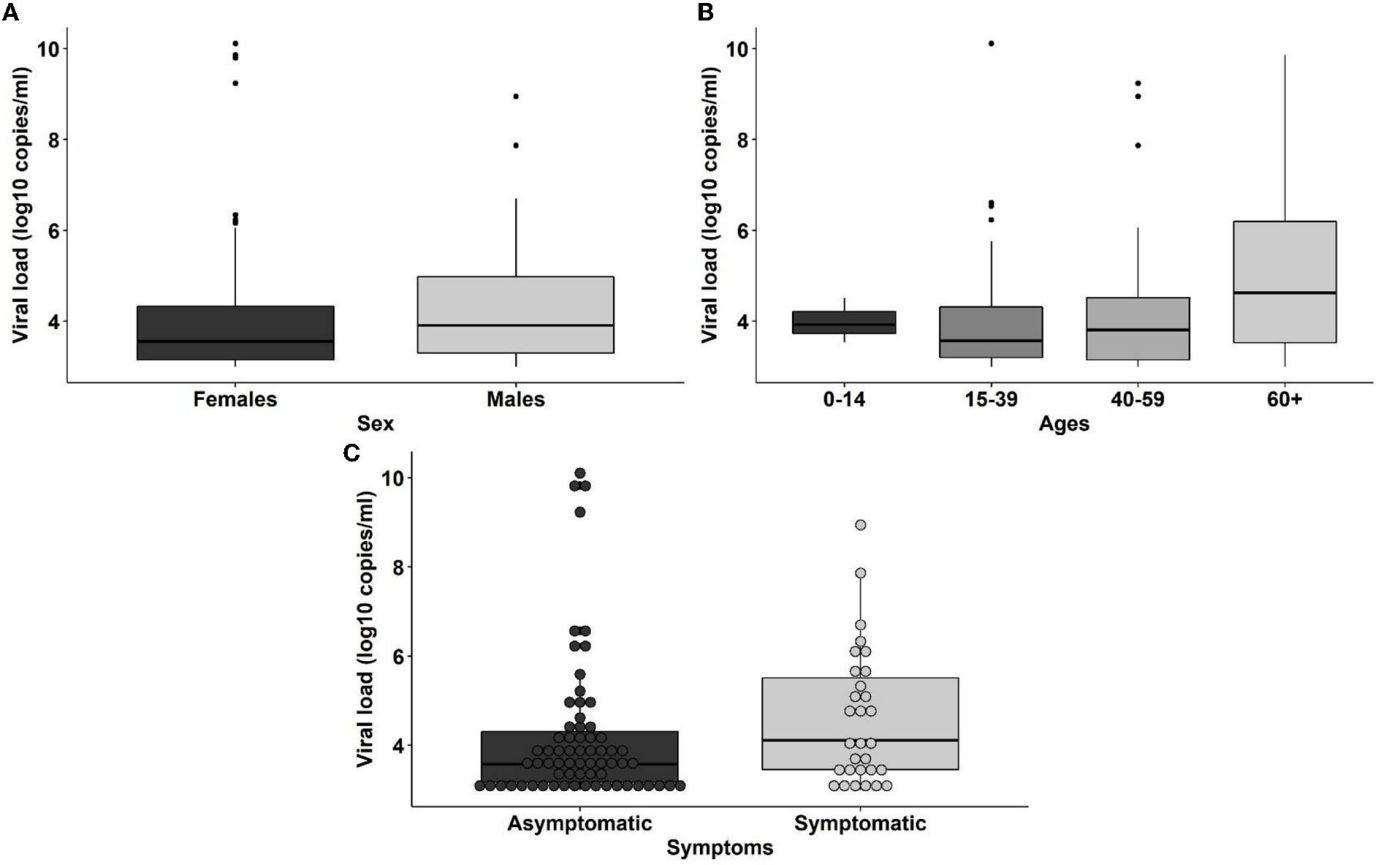
Distribution of viral loads among sexes **(A)**, age ranges (0–14: children, 15–39: young adults, 40–59: adults, 60+: elders) **(B)** and coronavirus disease 2019 (COVID-19) symptoms status **(C)** of the individuals.

The SARS-CoV-2 viral load distribution between age groups, sex, and COVID-19 symptoms status is presented in Figure 2. We found no significant difference in the viral loads among any of those variables (*p*-value > 0.05). There are 6 individuals with viral loads bigger than 10^8^ copies/ml, representing 6.2% of the SARS-CoV-2 positive population (6/97; 95% *CI*: [2.54–13.50%]) (Figure 2, Table 2) that are considered SARS-CoV-2 super spreaders (23). No association was found between the super spreader condition with sex, age, or presence of symptoms (Table 2).

**Table 2:** Demographic and clinical information from the super-spreaders.

**Super-spreader**	**Age**	**Sex**	**Location**	**Symptoms**	**Viral Load (log_10_ copies/mL)**
1	30	Female	Quininde	Asymptomatic	10.105
2	68	Male	Quininde	Asymptomatic	9.854
3	68	Female	Esmeraldas	Asymptomatic	9.791
4	43	Female	Esmeraldas	Asymptomatic	9.234
5	59	Male	Esmeraldas	Symptomatic	8.947
6	49	Male	Esmeraldas	Symptomatic	7.866

### Seroprevalence of anti-SARS-CoV-2 IgG

A total of 1,250 serological tests were applied across the same population considered for the RT-qPCR testing. The overall seroprevalence in Esmeraldas province was 11.68% (95% *CI*: [9.98–13.62%]), with 146 positive IgG tests (Table 1; Supplementary Figure 2). The seropositive population was distributed as follows (Supplementary Figure 2): 58 men and 88 women, average age: 45.25 ± 1.48 years old. Significant differences were found in the seroprevalence of anti SARS-CoV-2 IgG between men (9.53%, 58/608, 95% *CI*: [7.38–12.23%]) and women (13.71%, 88/642, 95% *CI*: [11.19–16.67%]; *p*-value <0.05), as well as in the seroprevalence for each age group: children (18.6%, 13/70, 95% *CI*: [10.64–30.02%]), young adults (7.2%, 40/553, 95% *CI*: [5.28–9.80%]), adults (13.2%, 57/431, 95% *CI*: [10.25–16.88%]), and elders (18.4%, 36/196, 95% *CI*: [13.35–24.66%]).

For the rural communities from Atacames, Muisne, Rio Verde, and Quininde, 60 anti-SARS-CoV-2 IgG positive individuals out of 475 ones tested were found, yielding a seroprevalence of 12.63% (95% *CI*: [9.85–16.04%]). For the urban communities from Esmeraldas canton, 86 anti-SARS-CoV-2 IgG positive individuals out of 775 ones tested were found, resulting in a seroprevalence of 11.10% (95% *CI*: [9.02–13.57%]). Those values were not statistically different (*p*-value > 0.05).

Regarding the anti-SARS-CoV-2 IgG seroprevalence for the different cantons (Table 1), Atacames had the highest value of 24.47% (23/94, 95% *CI*: [16.44–34.61]), followed by Quininde with 12.21% (32/262, 95% *CI*: [8.62–16.95]), Esmeraldas with 11.10% (86/775, 95% *CI*: [9.02–13.57]), Muisne with 6.78% (4/59, 95% *CI*: [2.19–17.27]), and Río Verde with 1.67% (1/60, 95% *CI*: [0.09–10.14]). Significant differences were found between those values (*p*-value <0.01).

## Discussion

After more than 2 years of the COVID-19 pandemic, the epidemiological information available from low and middle-income countries like those in Latin America is still scarce compared to high-income countries. Moreover, this information is mostly coming from governments' reports that are mainly focused in hospitalized patients as it has been shown for Ecuador (5, 7, 10, 23–27). According to the few scientific reports available detailed in Table 3, SARS-CoV-2 community transmission was happening in rural and remote areas of Ecuador (25–28) during the first semester of the COVID-19 pandemic. Unfortunately, not enough testing capacity was installed across the country (5, 7, 10) and no official information about the epidemiological situation of SARS-CoV-2 among rural and remote vulnerable populations like ethnic minorities was available (10, 25, 27, 28). For instance, the 1,259 samples collected for SARS-CoV-2 testing within a week period in October 2020 by our medical brigades represented 12.3% of the total RT-qPCR performed at Esmeraldas province since the COVID-19 outbreak in February 2020 (10). In this context, the present study addresses the impact of the first wave of COVID-19 pandemic in historically neglected population like the Afro-Ecuadorians from Esmeraldas province (29).

**Table 3:** Comparison of SARS-CoV-2 infection rate by RT-qPCR and anti-SARS-CoV-2 Ig seroprevalence for surveillance studies performed in Ecuador during the COVID-19 pandemics (NA: Not available).

**Location**	**Number of samples**	**SARS-CoV-2 infection rate (%)**	**anti-SARS-CoV-2 IgG seroprevalence (%)**	**Reference**
Esmeraldas	1,259	7.72	11.68	Present study
Manabi	4,003	16.13	NA	25,35
Santa Elena	673	NA	45.02	28
Galapagos	2,480	5.85	3.30	Manuscript under preparation.
Amazonia	769	49.16	NA	27,36

We found an overall SARS-CoV-2 infection rate of 7.71% in Esmeraldas province, with values as high as 12.17% for Quininde canton, confirming active SARS-CoV-2 community transmission as the population targeted was community-dwelling non-hospitalized individuals. This finding confirms SARS-CoV-2 community transmission across rural and remote regions of Ecuador like Manabi, Santa Elena, the Amazonia, and Galapagos Islands, as it is shown in Table 3, where high SARS-CoV-2 infection rates were also found within community-dwelling population (25, 28). Despite the active transmission described from the SARS-CoV-2 RT-qPCR diagnosis displayed, the overall anti-SARS-CoV-2 IgG seroprevalence of 11.68%, with values as high as 24.47% for Atacames canton, also supports that SARS-CoV-2 community transmission has been happening for weeks before October 2020 in Esmeraldas. Moreover, considering that the serological test used on the study had a lack of sensitivity over 20% (24), the percentage of the population exposed to SARS-CoV-2 would be even higher than the observed. However, the SARS-CoV-2 infection rates were similar or higher than seroprevalence values, for instance at Quininde and Rio Verde cantons, indicating that COVID-19 outbreaks were probably a recent event prior to October 2020 in Esmeraldas.

We found striking results when comparing rural and urban communities. While a higher infection rate and seroprevalence would be expected at urban locations in Esmeraldas city (population of around 150,000 people), both parameters were slightly higher for the rural and relatively isolated cantons. However, this is due to the high values obtained for Atacames and Quininde, for either infection rates (7.45 and 12.17%) or seroprevalence (12.21 and 24.47%). Although the communities visited within those cantons were rural, both Atacames and Quininde have certain socioeconomics features that would potentially explain those results. On one hand, Quininde is less than 20 miles away from Santo Domingo de los Taschilas that it is the fourth capital province in population (over 250,000 people) in Ecuador, and also an important commercial and communication hub for the region. On the other hand, Atacames is the host of multiple hotel facilities as its popular beaches received thousands of visitors from the capital city of Quito and other places of Ecuador.

Although any particular trend regarding SARS-CoV-2 viral loads or attack rates associated with either sex, age, or symptoms status was found at our study population, 6 individuals had viral loads in the range from 10^8^ to 10^10^ viral copies/ml and could be considered SARS-CoV-2 as super spreaders, representing a striking 6.2% of the infected population (23). Although there are limitations associated to calculate the viral load based on *C*_t_ values representing all the viral genomic material on the sample, and infection of cell cultures is used for sample infectivity confirmation, it is a clear association between low *C*_t_ values (that mean high viral loads based on genomic material quantification) and infectivity (23). Those super spreader individuals were either completely asymptomatic or reported some minor mild symptoms at the time of sample collection. This finding is particularly worrying considering that we did not observe a strong adherence to neither mask use nor social distancing in the communities visited. The impact of the COVID-19 pandemic was terrific worldwide, but rural communities and ethnic minorities like the Afro-Ecuadorian people from Esmeraldas were even more exposed and in risk of having severe consequences from COVID-19 outbreaks during the first wave of COVID-19 pandemic. As demonstrated by other studies and ours, the conditions imposed by climate and poverty at rural settings in the Ecuadorian Coastal and Amazonian Regions make those communities prone to the spread of SARS-CoV-2 (27, 29–35), which remarks the necessity of the optimal implementation of control and prevention strategies in these neglected territories.

Due to the retrospective nature of this study, there was not a randomized sample collection to include a statistically representative population sampling for Esmeraldas region. This is a strong limitation in our work, as the bias on sample collection could mean that the results obtained were not truly representative of the COVID-19 epidemiological context in this region, but were limited to the communities selected. However, SARS-CoV-2 community transmission was confirmed either by RT-qPCR or serology at all the communities visited; moreover, considering that those communities were quite similar in terms of socio-economical features to most of the communities in Esmeraldas province, we suggest that SARS-CoV-2 community transmission across this region during the first wave of COVID-19 is a plausible conclusion of this surveillance study.

The findings presented in this study of active community transmission and super spreading events among community-dwelling individuals in rural and remote locations would be useful for future pandemics. COVID-19 control and prevention strategies have to focus not only on hospitalized and symptomatic individuals but also including community-dwelling individuals at locations where outbreaks are suspected.

## Data availability statement

The original contributions presented in the study are included in the article/Supplementary material, further inquiries can be directed to the corresponding author/s.

## Ethics statement

The studies involving human participants were reviewed and approved by Hospital San Francisco de Quito. Written informed consent to participate in this study was provided by the participants' legal guardian/next of kin.

## UDLA COVID-19 team

Tatiana Jaramillo, Daniela Santander Gordon, Gabriel Alfredo Iturralde, Julio Alejandro Teran, Karen Marcela Vasquez, Jonathan Dario Rondal, Genoveva Granda, Ana Cecilia Santamaria, Cynthia Lorena Pino, Oscar Lenin Espinosa, Angie Buitron, David Sanchez Grisales, Karina Beatriz Jimenez, Vanessa Bastidas, Dayana Marcela Aguilar, Ines Maria Paredes, Christian David Bilvao, Sebastian Rodriguez Pazmiño, Juan Carlos Laglaguano, Henry Herrera, Pablo Marcelo Espinosa, Edison Andres Galarraga, Marlon Steven Zambrano-Mila, Ana Maria Tito, Nelson David Zapata.

## Author contributions

All authors listed have made a substantial, direct, and intellectual contribution to the work and approved it for publication.

## Funding

This study was supported by Fondo Salvar Vidas (Banco de Guayaquil) and Universidad de Las Américas.

## Conflict of interest

The authors declare that the research was conducted in the absence of any commercial or financial relationships that could be construed as a potential conflict of interest.

## Publisher's note

All claims expressed in this article are solely those of the authors and do not necessarily represent those of their affiliated organizations, or those of the publisher, the editors and the reviewers. Any product that may be evaluated in this article, or claim that may be made by its manufacturer, is not guaranteed or endorsed by the publisher.

## Footnotes

^1^https://www.salud.gob.ec/actualizacion-de-casos-de-coronavirus-en-ecuador/.
