# Supplementary material for: Sustained COVID-19 community transmission and potential super spreading events at neglected afro-ecuadorian communities assessed by massive RT-qPCR and serological testing of community dwelling population
Source: Front Med (Lausanne). 2022 Aug 18;9:933260. doi: 10.3389/fmed.2022.933260 (PMC9433781; doi:10.3389/fmed.2022.933260)
Supplement: Supplementary file 1 [file Table_1.pdf]

Characteristics of Esmeraldas study population: Number of samples collected		
**Location**	Rio Verde	65
	Quininde	263
	Muisne	59
	Esmeraldas	778
	Atacames	94
**Sex**	Males	614
	Females	645
**Age Groups**	0-14	71
	15-39	556
	40-59	433
	60+	199
**Result**	Positive	97
	Negative	1162

Supplementary Table 1: Characteristics of population from Esmeraldas included on this SARS-CoV-2 surveillance study. Number of samples collected by different groups and locations are displayed.
