# Supplementary material for: Sustained COVID-19 community transmission and potential super spreading events at neglected afro-ecuadorian communities assessed by massive RT-qPCR and serological testing of community dwelling population
Source: Front Med (Lausanne). 2022 Aug 18;9:933260. doi: 10.3389/fmed.2022.933260 (PMC9433781; doi:10.3389/fmed.2022.933260)
Supplement: Supplementary file 2 [file Image_1.pdf]

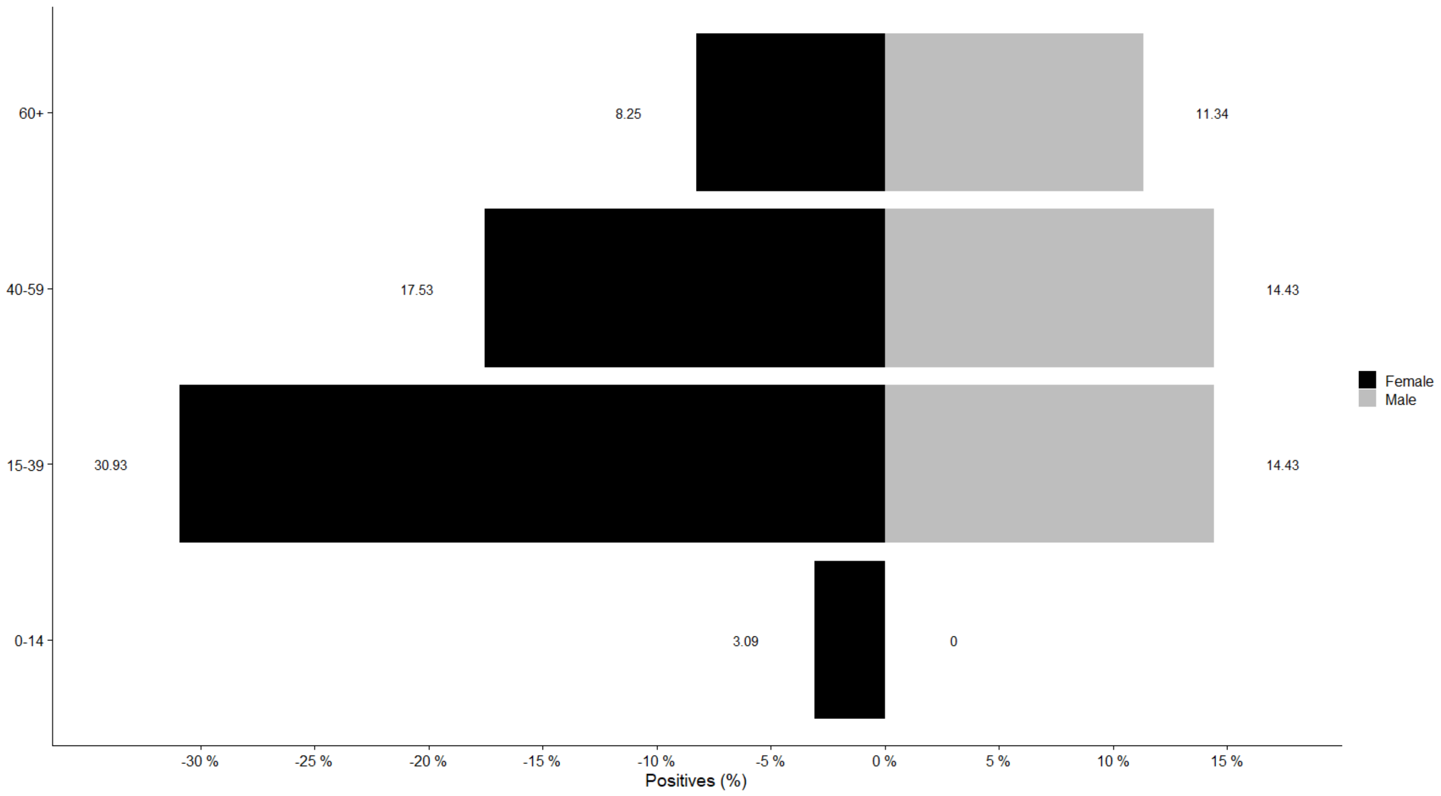

Supplementary Figure 1: Distribution of SARS-CoV2 positive cases according to sex and age in Esmeraldas by RT-qPCR testing.
