# Supplementary material for: Sustained COVID-19 community transmission and potential super spreading events at neglected afro-ecuadorian communities assessed by massive RT-qPCR and serological testing of community dwelling population
Source: Front Med (Lausanne). 2022 Aug 18;9:933260. doi: 10.3389/fmed.2022.933260 (PMC9433781; doi:10.3389/fmed.2022.933260)
Supplement: Supplementary file 3 [file Image_2.pdf]

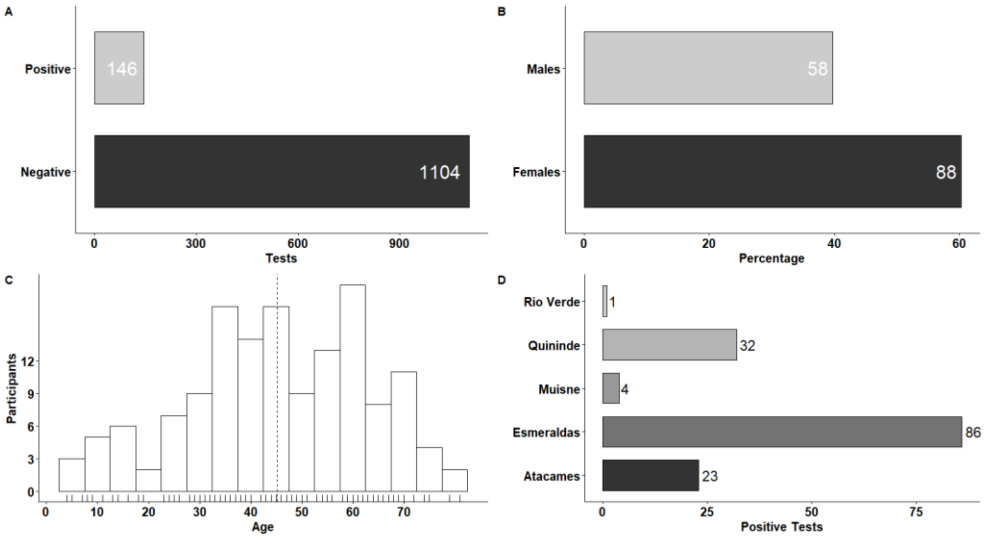

Supplementary Figure 2. Characteristics of the population included in the anti-SARS-CoV-2 IgG surveillance in Esmeraldas. A. Distribution of IgG positive and negative individuals included in the study. Distribution of IgG positive individuals by sex (B), age (C) and canton (D).
